# Minocycline Suppresses Interleukine-6, Its Receptor System and Signaling Pathways and Impairs Migration, Invasion and Adhesion Capacity of Ovarian Cancer Cells: In Vitro and In Vivo Studies

**DOI:** 10.1371/journal.pone.0060817

**Published:** 2013-04-08

**Authors:** Parvin Ataie-Kachoie, David L. Morris, Mohammad H. Pourgholami

**Affiliations:** Department of Surgery, University of New South Wales, St George Hospital, Sydney, New South Wales, Australia; China Medical University, Taiwan

## Abstract

Interleukin (IL)-6 has been shown to be a major contributing factor in growth and progression of ovarian cancer. The cytokine exerts pro-tumorigenic activity through activation of several signaling pathways in particular signal transducer and activator of transcription (STAT3) and extracellular signal-regulated kinase (ERK)1/2. Hence, targeting IL-6 is becoming increasingly attractive as a treatment option in ovarian cancer. Here, we investigated the effects of minocycline on IL-6 and its signaling pathways in ovarian cancer. *In vitro*, minocycline was found to significantly suppress both constitutive and IL-1β or 4-hydroxyestradiol (4-OH-E2)-stimulated IL-6 expression in human ovarian cancer cells; OVCAR-3, SKOV-3 and CAOV-3. Moreover, minocycline down-regulated two major components of IL-6 receptor system (IL-6Rα and gp130) and blocked the activation of STAT3 and ERK1/2 pathways leading to suppression of the downstream product MCL-1. In female nude mice bearing intraperitoneal OVCAR-3 tumors, acute administration (4 and 24 h) of minocycline (30 mg/kg) led to suppression of IL-6. Even single dose of minocycline was effective at significantly lowering plasma and tumor IL-6 levels. In line with this, tumoral expression of p-STAT3, p-ERK1/2 and MCL-1 were decreased in minocycline-treated mice. Evaluation of the functional implication of minocycline on metastatic activity revealed the capacity of minocycline to inhibit cellular migration, invasion and adhesion associated with down-regulation of matrix metalloproteinases (MMP)-2 and 9. Thus, the data suggest a potential role for minocycline in suppressing IL-6 expression and activity. These effects may prove to be an important attribute to the upcoming clinical trials of minocycline in ovarian cancer.

## Introduction

Ovarian cancer is a highly lethal gynecological malignancy for which overall prognosis has remained poor over the past few decades [1]. To explain the underlying molecular mechanisms involved in the development of this deadly malignancy a number of theories have been proposed so far [1]. Convincing data support the involvement of the inflammatory stromal microenvironment, caused by over-expression of cytokines and chemokines in promoting ovarian tumorigenesis and cancer progression [2]. In particular, IL-6 produced by tumor and/or activated immune cells has been postulated to be a key functional cytokine that alters tumor cells behaviors through complex mechanisms [3]. IL-6 is expressed by primary human ovarian surface epithelial cells and has been detected profoundly higher in tumor tissue [4], plasma and malignant ascites of ovarian cancer patients [5]. IL-6 expression in the ovarian tumor microenvironment can impact host immune defense mechanisms as well as tumor cell growth, proliferation, differentiation and angiogenesis [6]. It also induces metastasis through up-regulation of cancer cells adhesion and invasion capacity [7]. Furthermore, IL-6 influences clinical disease status and prognosis, by conferring resistance to conventional therapies [8], stimulating malignant ascites formation [9], and inducing symptoms such as anorexia, altered energy metabolism, fatigue and anemia [10]. Recent data evidence that blockade of IL-6 may offer a promising therapeutic strategy to improve the management of patients with ovarian cancer. Inhibition of IL-6 signaling has been shown to attenuate tumor growth and the apoptotic and metastatic events in ovarian cancer patients. It also resulted in prolonged periods of disease stabilization, reversal of chemoresistance and amplified host immunity in patients with chemoresistant recurrent ovarian cancer [3].

IL-6 transmits its signals through interacting with a receptor complex consisting of the ligand-binding glycoprotein termed IL-6R and the signal-transducing component gp130. There are two types of IL-6R, i.e., cell membrane IL-6 receptor (IL-6Rα) with low affinity that forms a complex with gp130 after binding with IL-6 to start the intracellular signal (classical signaling), and a soluble IL-6 receptor (sIL-6R) which binds with IL-6 and then with the membrane receptor β chain – gp130 leading to the signal transduction (trans-signaling) [11]. The signal transduction of IL-6 involves activation of several oncogenic pathways. In particular STAT3 is phosphorylated and activated in response to IL-6. After phosphorylation, STAT3 forms a dimer which is then translocated to the nucleus to regulate the expression of several genes leading to the induction of a series of events including tumor cell growth, survival and metastasis. In addition to STAT3, mitogen activated protein kinases (MAPK) cascade is also activated in response to IL-6 which leads to hyperphosphorylation of a series of proteins including ERK1/2 which in turn mediate activation of transcription factors with diverse effects on tumor cells including the induction of survival, migration and invasion capacity [12].

Minocycline (7-dimethylamino-6-desoxytertracycline) is a well-tolerated and safe antibiotic from the second-generation tetracycline family. Beside its broad-spectrum antibiotic activity, minocycline is also recognized as an anti inflammatory agent. It is used clinically in the treatment of diseases with inflammatory background such as acne [13] and bullous pemphigoid [14]. Moreover, minocycline has been proved effective in the treatment of autoimmune diseases such as rheumatoid arthritis [15] and scleroderma [16]. The potent anti-inflammatory and cell modulatory activities of minocycline are thought to be mediated through inhibition of proinflammatory cytokines such as tumor necrosis factor α [17] and IL-1β [18]; and suppression of MMPs [19]. Of particular interest, are data showing suppression of IL-6 by minocycline in monocytes [20] and central nervous system resident or infiltrating cells [21]. This inhibitory effect has also been reported to the IL-6 surge observed in neuropathic pain [22]. Experimental data using various carcinoma cell lines and animal models has also shown that minocycline and a number of other tetracyclines and their chemically modified derivatives may inhibit tumor growth and metastasis by suppressing matrix metalloproteinases and by a direct effect on cell proliferation. The antitumor effects of these agents have so far been documented in pre-clinical models of leukemia [23], melanoma [24], renal, prostate [25] and breast cancer [26]. Along this line, we have recently communicated the preliminary results showing that minocycline inhibits growth of human ovarian cancer xenografts [27], [28] and also suppresses ovarian cancer induced malignant ascites formation [28]. The recognition of these properties along with the favorable pharmacological and physiochemical profile of minocycline including high lipophilic property, complete oral absorption [29], a long half life, clinically desired pharmacokinetic characteristics [30] and a good safety profile (average tolerated oral dose 400 mg/day) [31] led us to design the current study to profile this drug with respect to its effects on IL-6 and IL-6 signaling pathways in ovarian cancer. Herein, we present our *in vitro* results showing the ability of minocycline to reduce both constitutive and stimulated (either by IL-1 ß or 4-OH-E2) IL-6 expression in ovarian cancer cells. Moreover, we have shown that minocycline suppresses IL-6 receptor system (IL-6R and gp130), signaling pathways (STAT3 and ERK1/2) and downstream target MCL-1 in these cells. Additionally, we have demonstrated that minocycline interferes with the metastatic potentials of ovarian cancer cells which was associated with suppression of MMP-2 and MMP-9. This is followed by our *in vivo*-based investigation revealing for the first time the effects of minocycline to reduce both plasma and tumoral IL-6 expression along with down-regulation of tumoral p-STAT3, p-ERK1/2 and MCL-1 in an experimental model of ovarian cancer in mice.

## Materials and Methods

### Ethics Statement

All animal works have been conducted according to University of New South Wales Animal Care and Ethics Committee (ACEC) guidelines. All procedures carried out on mice were in strict accordance with the protocol approved by ACEC (approval number: 9/23B) and all efforts were made to minimize suffering.

### Chemicals and Antibodies

Unless otherwise stated, all drugs and chemicals used in this study were obtained from Sigma-Aldrich (Australian subsidiary, Sydney, Australia). The following primary antibodies were used throughout this study: rabbit polyclonal antibodies specific for IL-6Rα, gp130, Tyr^705^-p-STAT3, STAT3, Mcl-1, p-p44/42 MAPK (p-ERK1/2), p44/42 MAPK (ERK1/2) (Cell Signaling Technology, Sydney, Australia), MMP-2 and MMP-9 (Santa Cruz, Sydney, Australia) and mouse Monoclonal anti-β-actin (R&D Systems, Inc., Sydney, Australia). Secondary antibodies were goat anti rabbit or anti mouse immunoglobulin G conjugated with horseradish peroxidise (Santa Cruz Biotechnology, Sydney, Australia).

### Cell Culture

The human ovarian cancer cell lines; OVCAR-3, SKOV-3 and CAOV-3 cells, were obtained from American Type Culture Collection (ATCC, Manassas, VA). Cells were maintained in RPMI 1640 medium with 2 mM l-glutamine, 2 g/L sodium bicarbonate, 4.5 g/L glucose, 10 mM HEPES, 1 mM sodium pyruvate (Invitrogen, Sydney, Australia) supplemented with 10% heat inactivated fetal bovine serum (FBS) and penicillin–streptomycin (50 U/ml) at 37°C in a humidified atmosphere containing 5% CO2.

### *In vivo* Experiments

Female nude athymic Balb C nu/nu mice (6 weeks old) were purchased from Biological Resources (Faculty of Medicine, University of New South Wales). The mice were housed and maintained in laminar flow cabinets under specific pathogen-free conditions in facilities approved by the University of New South Wales Animal Care and Ethics Committee (ACEC). All procedures carried out on mice were in strict accordance with the protocol approved by ACEC (approval number: 9/23B) and all efforts were made to minimize suffering. Briefly, 10×10^6^ log-phase growing OVCAR-3 cells suspended in 0.5 ml phosphate-buffered saline (PBS) were injected intraperitoneally (i.p.) to each mouse. On day 28 after cell inoculation, mice were randomly assigned to one of the treatment or control groups. Minocycline was dissolved in sterile normal saline (0.6 mg/ml). Mice were injected i.p. with a single dose of minocycline (30 mg/kg). Control group received sterile normal saline instead. At the end of treatment period (4 or 24 h), blood was collected through cardiac puncture, animals were euthanized using Lethabarb R (100 mg/kg) i.p. injection (VIRBAC, Sydney, Australia) and tumors were immediately dissected and preserved in -80°C for western blot analysis.

### Immunocytochemistry Staining

Cells were seeded onto sterilized glass cover slips. Then they were treated with minocycline for 24 h, washed with PBS and fixed with 100 µl per slide of cooled 95% ethanol, 5% glacial acetic acid for 10 min. Fixed cells were then washed and incubated in 0.3% Tween 20 for 20 min, washed with PBS, blocked with 1% BSA, incubated with primary antibodies in 1% BSA followed by Alexafluor-conjugated secondary antibodies in 1% BSA. Cell nuclei were stained with propidium iodide (PI) (1∶2000 dilution) for 1 min before cover slips were mounted on glass slides using glycerol, and analyzed for protein expression using Olympus IX71 laser scanning microscopy with 60× oil immersion lens.

### Enzyme-Linked Immunosorbent Assay (ELISA) for IL-6

*In vitro*, cells were seeded in 6-well plates in 10% FBS media for 24 h to complete attachment. Then they were either stimulated with IL-1β (10 ng/ml) or 4-OH-E2 (50 µg/ml) or non-stimulated with or without pretreatment with minocycline. IL-6 produced in the culture medium was quantified using the IL-6 specific ELISA kit according to the manufacturer’s instructions (Biolegend Inc., San Diego, CA). The attached cells were counted to normalize IL-6 concentrations against number of cells. Quantitative determination of IL-6 content of the plasma from *in vivo* study was also carried out using the same ELISA kit.

### Western Blot Analysis

To examine the effect of minocycline on cellular expression of gp130, IL-6Rα, p-STAT3, STAT3, Mcl-1, p-ERK, ERK, MMP-2 and MMP-9, western blot analysis was performed according to standard procedure. Briefly, cells were washed in ice-cold PBS and extracted for 30 min with a buffer containing 50 mM Tris-HCl, pH 7.5, 140 mM NaCl, 5 mM EDTA, 5 mM NaN3, 1% Triton X-100, 1% NP-40, 1 mM EGTA,10% phosphatase inhibitor and protease inhibitor cocktail. Lysates were cleared by centrifugation at 13,000 ×*g* for 30 min and protein concentrations were determined using Bio-red protein assay. Equivalent amounts of whole cell extracts were resolved by SDS- polyacrylamide gel electrophoresis and transferred onto a polyvinylidene difluoride membrane (Millipore Corporation, MA, USA). The membranes were then probed with specific antibodies. Immune-complexes were detected using horseradish peroxidase conjugated with either anti-mouse or anti-rabbit followed by chemiluminescence detection (Perkin Elmer Cetus, Foster City, CA, USA). To demonstrate equal protein loading, blots were stripped and reprobed with a specific antibody recognizing β-actin.

### Transwell Migration and Invasion Assay

Cell migration and invasion were determined using a 24-well Transwell system with polycarbonate membranes of 8 mm pore size **(**Life Technologies, Vic, Australia). Briefly, 1×10^3^ cells were seeded in 0.1% BSA RPMI medium containing varying concentration of minocycline in the upper chamber (normal chamber for migration assay and matrigel-coated chamber for invasion assay). The lower chamber was filled with the same medium containing 1% FBS. After incubating for 18 hr at 37°C, cells in the upper chamber were carefully removed with a cotton swab and the cells that had traversed to reverse face of the membrane were fixed in methanol, stained with Giemsa solution. For each replicate (n = 3), migration or invasion of the cells was quantified by counting the stained cells (cells per five fields) under inverted microscope.

### Cell Adhesion Assay

The assay was performed as previously described with minor modifications [32]. Briefly, SKOV-3 cells were grown to 70% confluence, serum-depleted, and then treated with varying concentrations of minocycline (0–100 µM) for 18 h. Cells were removed using nonenzymatic detachment solution, washed twice, resuspended in RPMI without serum containing different concentrations of minocycline (0–100 µM), and plated at a density of 1×10^4^ cells/well onto 96-well plates coated with collagen IV. After 30 min incubation at 37°C, the medium was removed, and plates were washed twice in PBS. Cells were then fixed with methanol and stained with 0.1% crystal violet solution, gently washed 3 times with PBS, and crystal violet-solubilized using acetic acid/methanol/water (10∶30∶60), and absorbance was read at 595 nm.

### Statistical Analysis

All statistical analyses were done with Graph Pad Prism software version 5.0 (CA, USA). Data are presented as mean ± SD. The student *t*-test was used to compare two independent groups means. One way analysis of variance (ANOVA) was used to determine the statistical differences between more than two groups and a two-way repeated measures ANOVA was used to evaluate time and treatment interaction effects for the dependent variable IL-6; a significant interaction was interpreted by a subsequent simple-effects analysis with Bonferroni correction. Statistical significance was established at the *p*<0.05 level.

## Results

### Minocycline Decreases Constitutive Expression of IL-6 in Ovarian Cancer Cells

**Figure 1:**
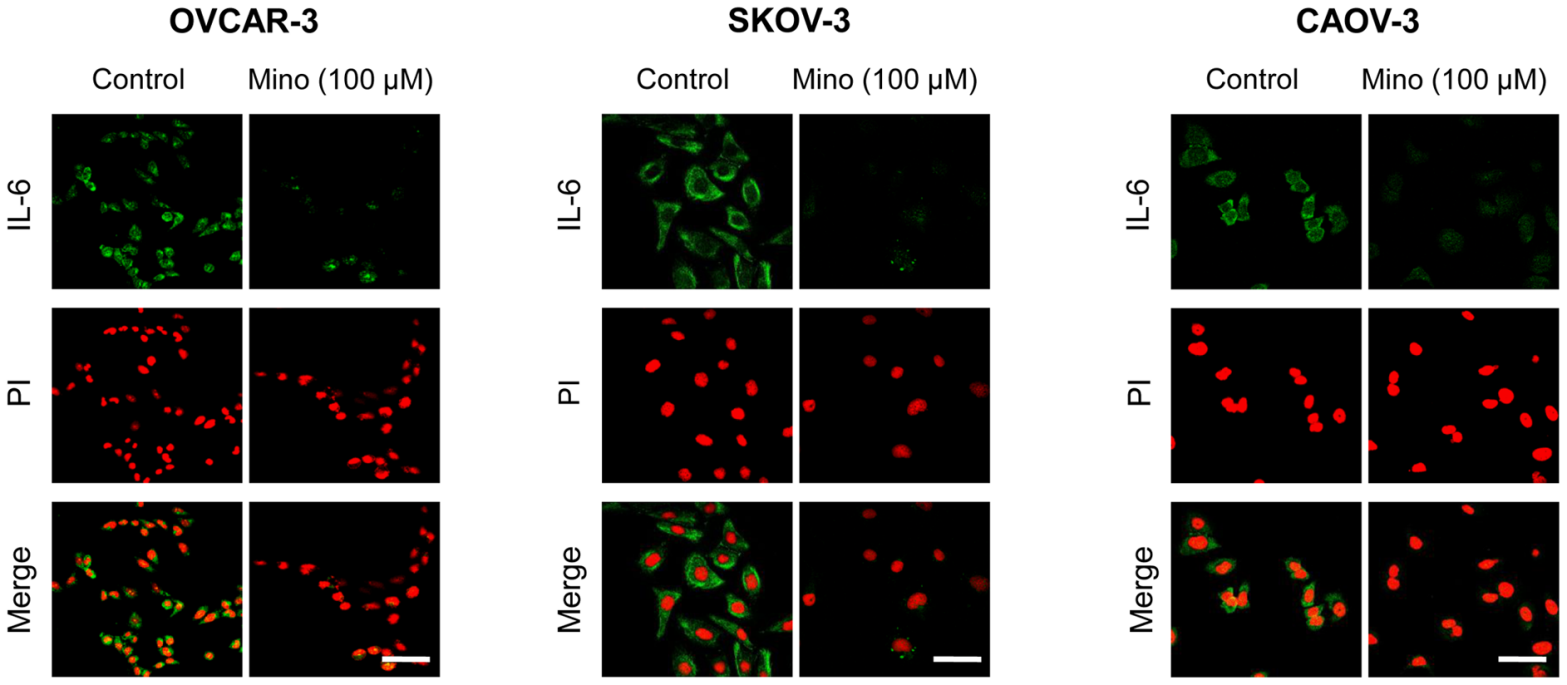
Minocycline decreases constitutive expression of IL-6 in ovarian cancer cell lines. Representative confocal images of IL-6 (green) in OVCAR-3, SKOV-3 and CAOV-3 cells under control conditions and exposed to minocycline (100 µM) for 24 h. Cells were also stained with propidium iodide (red). Images were obtained at 60× magnification. The scale bars represent 20 µm.

**Figure 2:**
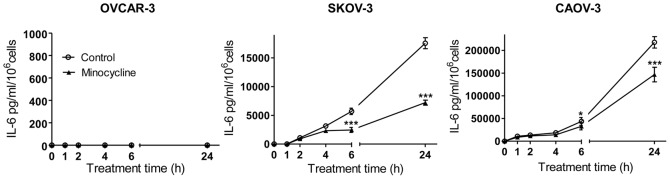
Effect of minocycline on basal IL-6 levels in media bathing ovarian cancer cells. Minocycline (100 µM) decreased IL-6 expression of SKOV-3 and CAOV-3 cells in a time-dependent pattern as analyzed by ELISA. The values shown are mean ± SD of data from three independent experiments (*p<0.05 and ***p<0.001 *vs.* control group).

To investigate the effect of minocycline on IL-6 expression in ovarian cancer cells, immunofluorescent staining was performed after 24 h treatment of OVCAR-3 (with low IL-6 expression), SKOV-3 (with medium IL-6 expression), and CAOV-3 (with high IL-6 expression) cell lines with minocycline (100 µM). The results demonstrated that minocycline significantly decreased the expression of IL-6 in all three ovarian cancer cell lines (Fig. 1). To quantify this effect, ELISA assay was used to determine the IL-6 levels in cell culture media bathing OVCAR-3, SKOV-3 and CAOV-3 cells treated with minocycline (100 µM) for 1, 2, 4, 6 and 24 h. As shown in Fig. 2, for OVCAR-3 cells IL-6 was not detectable in the media. However, minocycline treatment resulted in time-dependent decrease in IL-6 expression in both SKOV-3 and CAOV-3 cells which was significant at 6 h with percentage reduction relative to control: 55±5% for SKOV-3 (p<0.001), and 25±4.5% for CAOV-3 (p<0.05); and 24 h with percentage reduction relative to control: 60±7% for SKOV-3 (p<0.001) and 35±3.5% for CAOV-3 (p<0.001).

### Minocycline Blocks IL-6 Surge in Ovarian Cancer Cells Stimulated with IL-1β or 4-OH-E2

**Figure 3:**
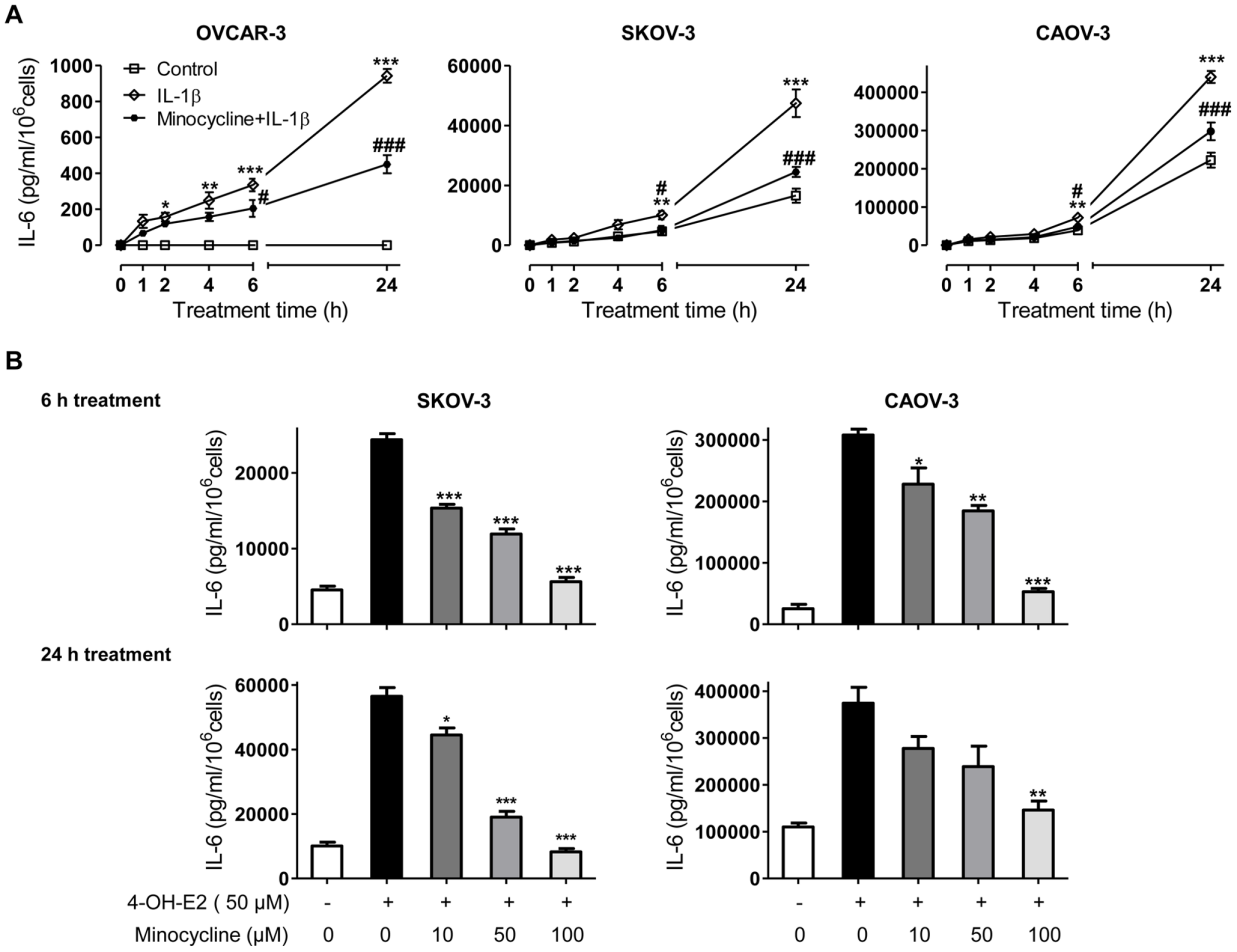
Minocycline inhibits IL-6 surge in stimulated ovarian cancer cell lines. (A) OVCAR-3, SKOV-3 and CAOV-3 cells were pre-treated with minocycline (100 µM) followed by stimulation with IL-1β (10 ng/ml) for different time points. Levels of IL-6 in the culture media were quantified by sandwich ELISA. Each data represent the mean ± SD from three independent experiments. (*p<0.05, **p<0.01 and ***p<0.001 *vs.* control group, ^#^p<0.05 and ^###^ *p*<0.01 *vs.* IL-1β-stimulated group). (B) OVCAR-3, SKOV-3 and CAOV-3 cells were pre-treated for 1 h with varying concentrations of minocycline (0–100 µM) followed by stimulation with 4-OH-E2 (50 µg/ml) for 6 and 24 h. Media were collected and IL-6 level was analyzed by ELISA. The values shown are mean ± SD of data from three independent experiments (*p<0.05, **p<0.01 and ***p<0.001 *vs.* 4-OH-E2-stimulated group).

IL-1β is a potent inducer of IL-6 in human cells [33]. It has been shown that both normal and malignant epithelial ovarian cells along with activated immune cells in the stroma produce IL-1β. Moreover, constitutive production of IL-1β by ovarian carcinoma cells stimulates production of cytokines such as IL-6 [34]. To simulate what happens in ovarian tumors, we used IL-1β (10 ng/ml) to induce IL-6 expression in ovarian cancer cells and then examined whether minocycline can block the surge in IL-6 expression. Using the standard ELISA kit, IL-6 concentrations in the media of OVCAR-3, SKOV-3 and CAOV-3 were measured at different time points post stimulation with IL-1β. It was observed that IL-6 concentration increased in a time-dependent manner in the presence of IL-1β in the media bathing all three cell lines. The maximum increase was observed at 6 h with the percentage increase relative to control of 335±15% for OVCAR-3 (p<0.001), 110±6% for SKOV-3 and 90±5% for CAOV-3 (p<0.01); and 24 h with the percentage raise relative to control of 940±13% for OVCAR-3, 185±7.8% for SKOV-3 and 100±5.4% for CAOV-3 (p<0.001) (Fig. 3A). Pretreatment of IL-1β-stimulated cells with minocycline (100 µM) led to significant inhibition of IL-6 surge at 6 h (the percentage decrease relative to IL-1β stimulated group: 40±7.3% for OVCAR-3, 50±4.5% for SKOV-3 and 35±6.2% for CAOV-3, p<0.05); and 24 h (the percentage inhibition relative to IL-1β stimulated group: 50±3.8% for OVCAR-3, 50±2.5% SKOV-3 and 30±5% for CAOV-3, p<0.001). To further confirm the IL-6 production modulating effect of minocycline, the effect of the drug was examined in 4-OH-E2-treated ovarian cancer cells. It is well documented that steroid hormones, particularly estrogens can modulate cytokine production through induction of IL-1β gene at transcription level [35]. 4-OH-E2 is the catechol metabolite of 17β-Estradiol which is the most biologically active ovarian estrogen. In ovarian cancer cells, 4-OH-E2 is a potent mitogenic substance [36] which induces other growth factors and carcinogenic pathways [37]. Here, OVCAR-3, SKOV-3 and CAOV-3 cells were treated with 4-OH-E2 (50 µg/ml) and IL-6 concentration was measured in cell culture media after 6 and 24 h. For OVCAR-3 cells, IL-6 was not detectable in the media up to 24 h post 4-OH-E2 treatment. However, 4-OH-E2 treatment resulted in a significant increase in IL-6 concentrations in both SKOV-3 and CAOV-3 cells media at 6 h (5 and 12 fold increase for SKOV-3 and CAOV-3, respectively) (p<0.001); and 24 h (6 and 3 fold increase for SKOV-3 and CAOV-3, respectively) (p<0.01) (Fig. 3B). As depicted in Fig. 3B, minocycline pretreatment of 4-OH-E2-stimulated cells resulted in concentration-dependent inhibition of IL-6 expression. At the concentration of 100 µM minocycline completely blocked IL-6 surge induced by 4-OH-E2 in both SKOV-3 and CAOV-3 cell lines.

### Minocycline Down-regulates IL-6R and gp130 Expression in Ovarian Cancer Cells

**Figure 4:**
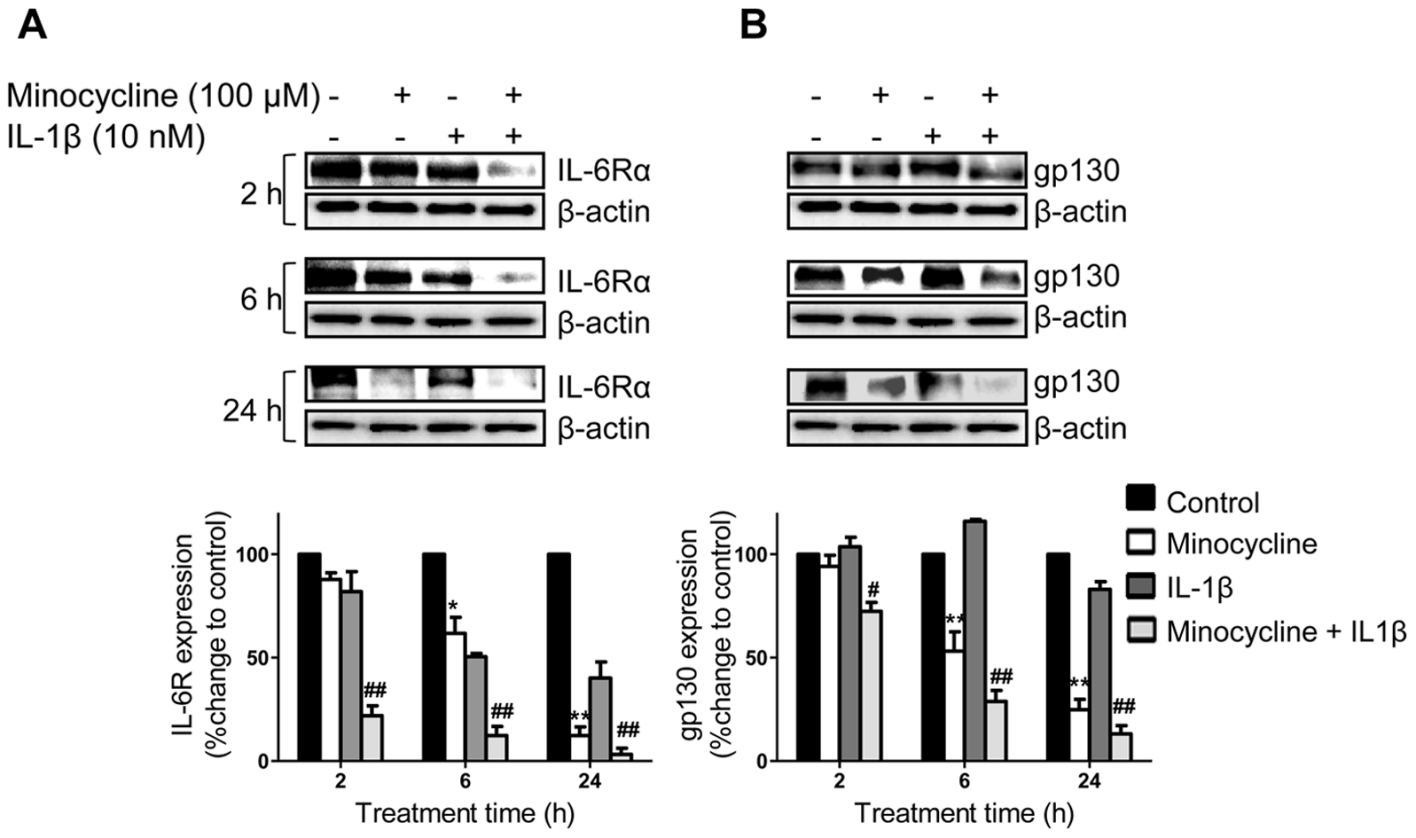
Effects of minocycline on IL-6Rα and gp130 expression in ovarian cancer cells. To detect expression levels of (A) IL-6Rα or (B) gp130, SKOV-3 cells were either stimulated with IL-1β (10 ng/ml) or non-stimulated, with or without pre-treatment with minocycline (100 µM) for different time points. Cell lysates were analyzed by immunoblotting for IL-6Rα, gp130 and β-actin antibodies. β-actin was the loading control. IL-6Rα and gp130 protein levels were normalized to β-actin and their relative differences with the corresponding controls are shown (*p<0.05 and **p<0.01 *vs.* control cells, ^#^ *p*<0.05 and^##^ *p*<0.01 *vs.* IL-1β treated cells).

To determine if minocycline affects IL-6 receptor system, western blot analysis were carried out to assess IL-6Rα and gp130 expression in SKOV-3 cells (IL-6Rα positive) after minocycline (100 µM) treatment with or without IL-1β stimulation. Exposure of both non-stimulated and IL-1β-stimulated SKOV-3 cells to minocycline led to significant down-regulation of IL-6Rα (Fig. 4A) and gp130 (Fig. 4B) in a time-dependant manner.

### Influence of Minocycline on STAT3 and ERK1/2 Phosphorylation and Mcl-1 Expression in IL-1β Stimulated and Non-stimulated Ovarian Cancer Cells

**Figure 5:**
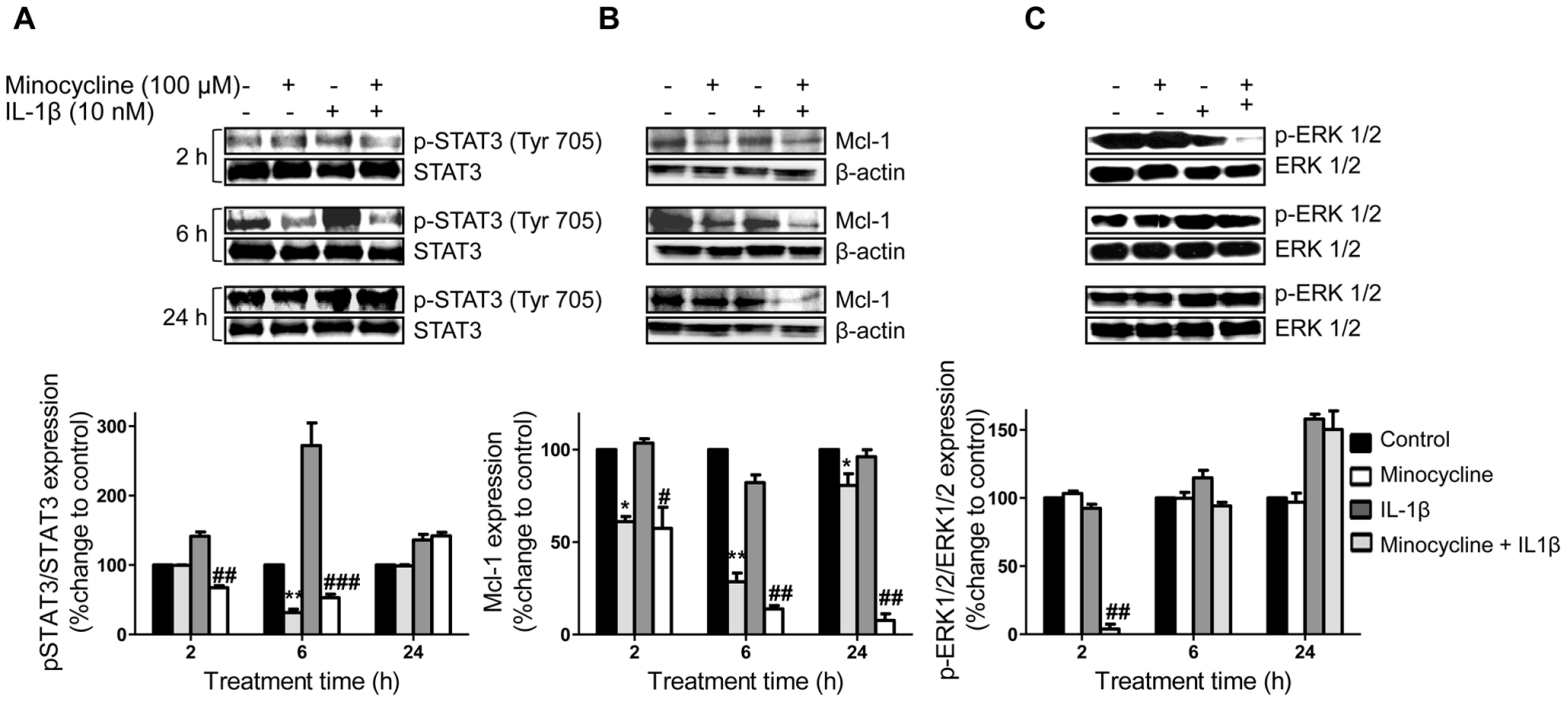
Influence of minocycline on p-STAT3, p-ERK1/2 and Mcl-1 expression in ovarian cancer cells. SKOV-3 cells were treated with minocycline (100 µM) with or without IL-1β (10 ng/ml) stimulation for different time points. The expression levels of (A) p-STAT3, STAT3; (B) Mcl-1 or (C) p-ERK1/2, ERK1/2 were estimated by western blot analysis. Densitometric analysis is expressed as mean ± SD intensity of optical density obtained by three independent experiments (*p<0.05, **p<0.01 and ***p<0.001 *vs.* control cells, ^#^ *p*<0.05, ^##^ *p*<0.01 *vs.* IL-1β treated cells).

To examine whether minocycline inhibits STAT3 phosphorylation in ovarian cancer, SKOV-3 cells expressing persistently activated STAT3 were treated with minocycline with or without IL-1β stimulation, for different time points. It was observed that minocycline treatment resulted in down-regulation of basal p-STAT3 in a time-dependent manner with the maximum inhibitory effect occurring at 6 h (2.5 fold decrease compared to control, p<0.01). IL-1β stimulation up-regulated the phosphorylation of STAT3 time-dependently with the maximum increase at 6 h (2.5 fold increase compared to control). Pretreatment of IL-1β-stimulated cells with minocycline inhibited STAT3 phosphorylation with the optimum effect at 6 h (5 fold decrease compared to IL-1β-stimulated group, p<0.001). However, this effect was reversible and the levels of p-STAT3 returned to control levels after 24 h of treatment. No significant changes were observed in total STAT3 levels (Fig. 5A).

MCL-1 is an apoptosis inhibitory protein downstream of IL-6/STAT-3 pathway. We thus evaluated the effect of minocycline on Mcl-1 in SKOV-3 cells under both normal and IL-1β-stimulated conditions. In non-stimulated SKOV-3 cells, minocycline treatment led to down-regulation of Mcl-1 protein starting at 2 h (1.5 fold decrease compared to control, p<0.05) with the maximum inhibition at 6 h (3 fold decrease compared to control, p<0.01). But eventually protein levels returned to normal control value by 24 h. Treatment of IL-1β-stimulated cells also led to a time-dependent inhibitory effect on Mcl-1 protein where the maximum inhibition could be observed at 24 h (6 fold decrease compared to IL-1β-stimulated group, p<0.01) (Fig. 5B).

In an attempt to determine whether minocycline inhibits MAPK pathway, the effects of minocycline on the phosphorylation of ERK1/2 in normal and IL-1β induced SKOV-3 cells were examined. As shown in Fig. 5C, no significant changes were observed in p-ERK1/2 protein levels of non-stimulated cells after minocycline treatment. IL-1β stimulation increased phorphorylation of ERK1/2 in a timely manner. Treatment with minocycline prior to IL-1β stimulation dramatically suppressed the phosphorylation of ERK1/2 at 2 h (8 fold decrease compared to IL-1β-stimulated group, p<0.01). However, p-ERK1/2 protein levels returned to normal control values by 24 h. Minocycline treatment did not alter the overall expression of ERK1/2 protein.

### Minocycline Inhibits Translocation of STAT3 to the Nucleus

**Figure 6:**
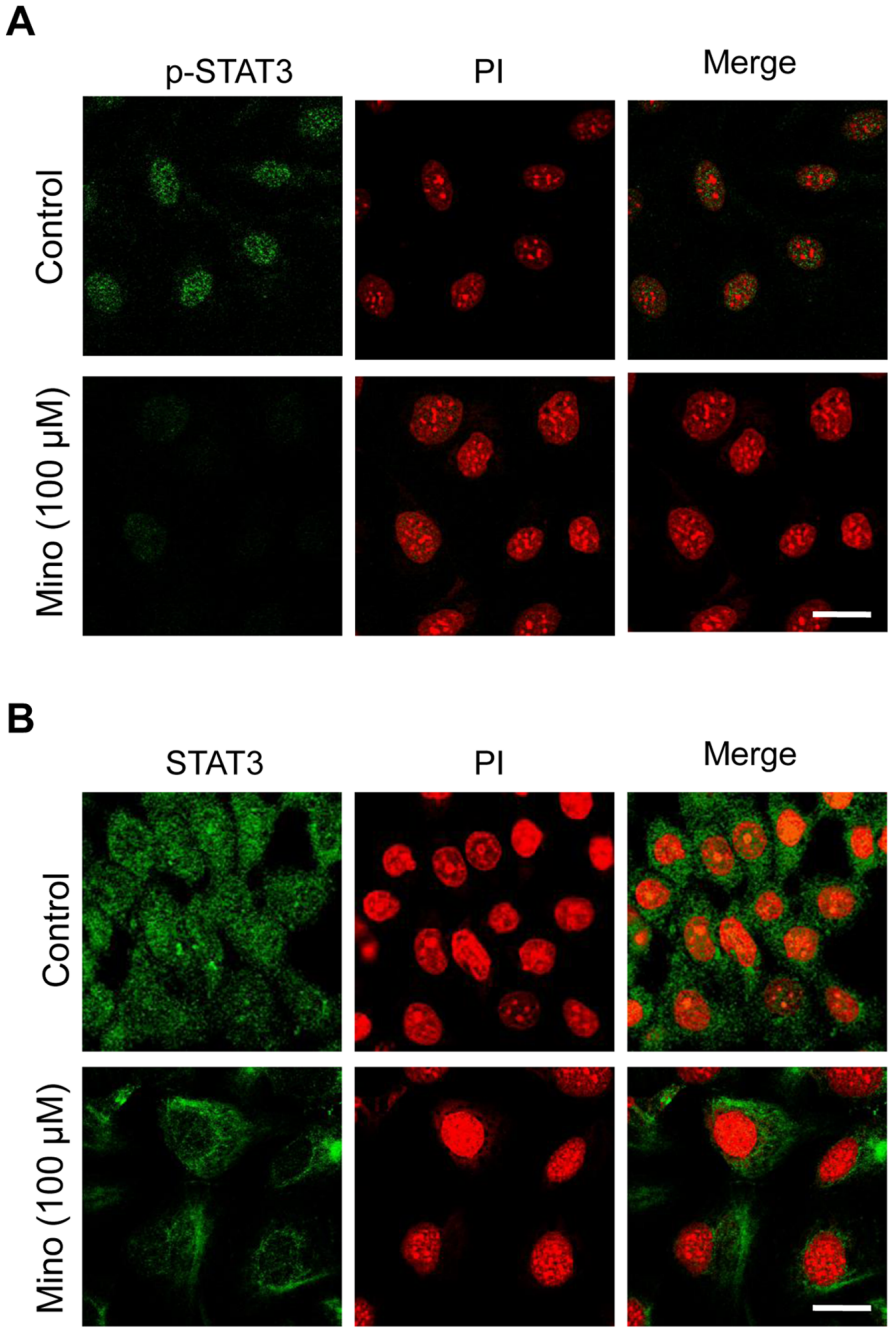
Effect of minocycline on STAT3 nuclear translocation. Confocal immunocytochemistry of (A) p-STAT3 and (B) STAT3 (green) in SKOV-3 cells treated with 100 µM minocycline in comparison with control cells. The nuclei are counterstained with propidium iodide (red). Images were obtained at 60× magnification. The scale bars represent 10 µm.

Following phosphorylation in cytoplasm, STAT3 translocates to the nucleus to render its transcriptional activity. Here, we used immunofluorescent confocal microscopy with antibodies that specifically bound to p-STAT3 (Tyr 705) or total STAT3 to confirm that minocycline blocks phosphorylation and nuclear translocation of STAT3. As depicted in Fig. 6A, minocycline (100 µM) treatment repressed phosphorylation of STAT3 in SKOV-3 cells which is in accordance with the western blot analysis results. Furthermore, nuclear translocation of STAT3 was inhibited in cells exposed to minocycline (100 µM) (Fig. 6B).

### Minocycline Attenuates Ovarian Cancer Cell Metastatic Potential Which is Associated with Decreased MMP-2 and MMP-9 Expression

**Figure 7:**
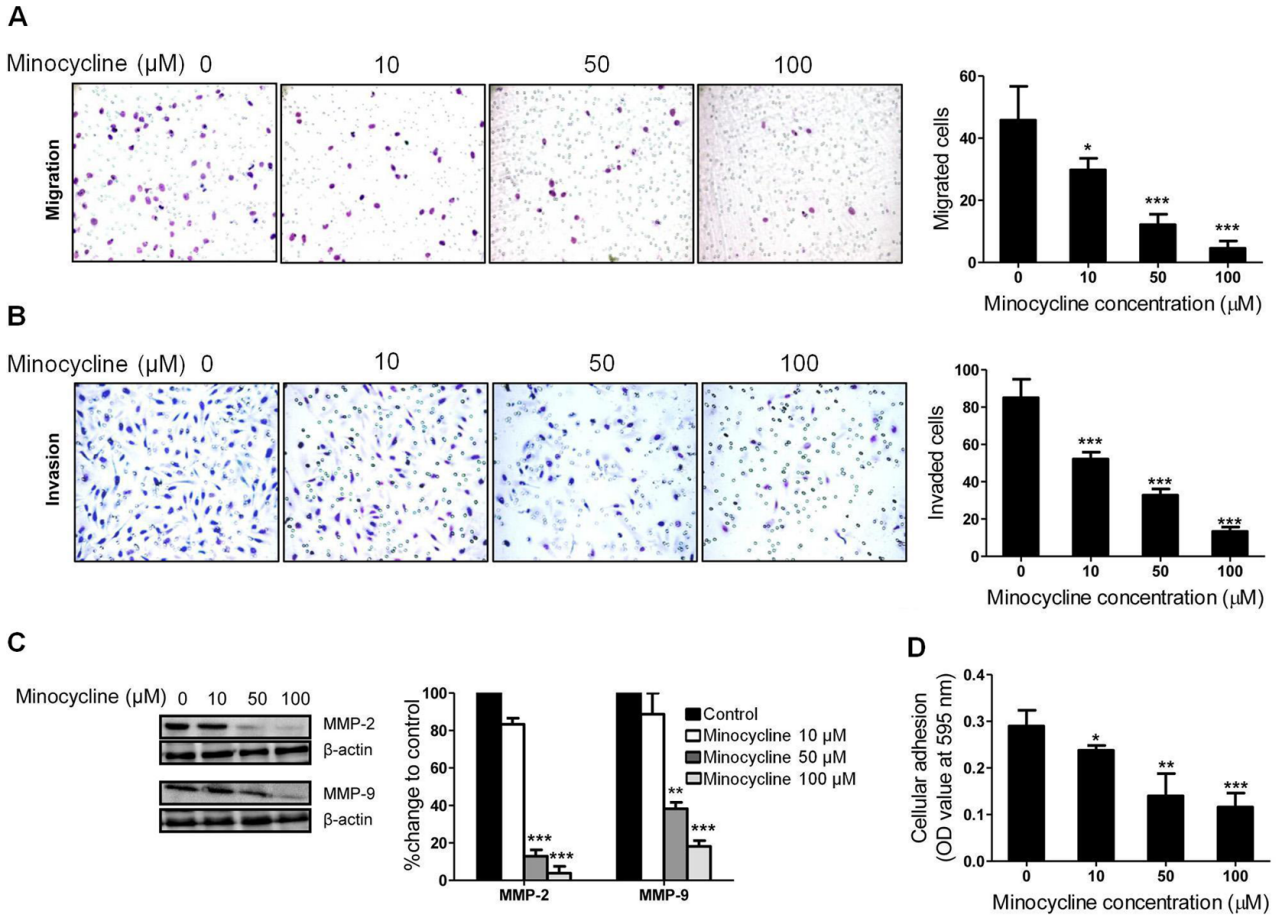
Minocycline inhibits metastatic capacity of SKOV-3 cells along with down-regulation of MMP-2 and MMP-9. The effects of minocycline on SKOV-3 (A) cell migration and (B) invasion was measured by Transwell assay. Minocycline was applied at different concentrations (0–100 µM) for 18 h. (C) Expression of MMP-2 and MMP-9 estimated by western blot analysis after 18 h treatment of SKOV-3 cells with varying concentration of minocycline. (D) Cell adhesion following 18 h exposure to varying concentrations of minocycline as described in MATERIAL AND METHODS (*p<0.05, **p<0.01 and ***p<0.001 *vs.* control).

IL-6 contributes to ovarian cancer metastasis via a complex multistep process involving the adhesion, migration and invasion of cancer cells [7]. We next performed Boyden chambers cell migration assay and matrigel invasion assay to determine if minocycline induced IL-6 suppression leads to inhibition of SKOV-3 cells metastatic activity. Treatment with minocycline (18 h) significantly attenuated the migration (Fig. 7A) and invasion (Fig. 7B) ability of SKOV-3 cells in a dose-dependent manner. Moreover, minocycline inhibited the capacity of SKOV-3 cells to adhere to wells coated with human collagen type IV dose-dependently (Fig. 7D).

Expression of MMPs, especially MMP-2 and MMP-9, can promote cell migration and invasion by the selective proteolysis of extracellular matrix components [38]. To study the effect of minocycline on MMP activity in ovarian cancer cells, we analyzed the expression of MMP-2 and MMP-9 in SKOV-3 cells after treatment with varying concentrations of minocycline for 18 h. As shown in Fig. 7C, minocycline decreased both MMP-2 and MMP-9 expression dose-dependently. These data suggest that the inhibition of the metastatic potential of ovarian cancer cells by minocycline is associated with decreased MMP-2 and MMP-9 expression.

### Minocycline Inhibits IL-6 in an Experimental Model of Ovarian Cancer in Mice

**Figure 8:**
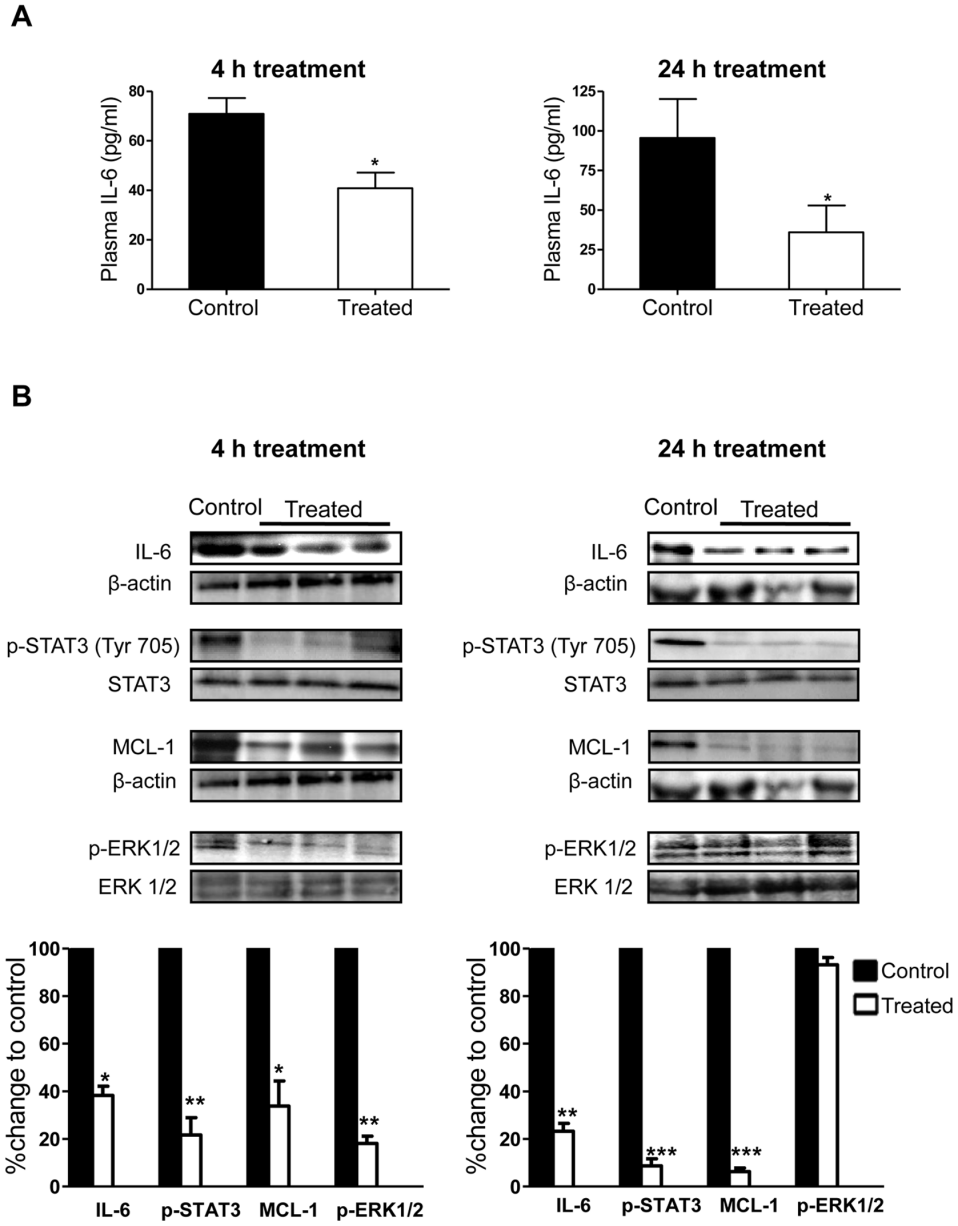
Suppression of IL-6 and its pathways by minocycline in mice bearing ovarian tumors. (A) IL-6 levels were determined by ELISA in the plasma of mice bearing i.p. OVCAR-3 tumors, 4 and 24 h post minocycline treatment (single dose 30 mg/kg; i.p.) (B) Tumors harvested at 4 and 24 h were examined by western blot analysis for the expression of IL-6, p-STAT3 (Tyr 705), MCL-1 and p-ERK1/2 protein. (*p<0.05, **p<0.01 and ***p<0.001 vs. control).

The ability of minocycline to inhibit IL-6 and its pathways in ovarian cancer cells *in vitro* was a good indication that it has the potential to affect the IL-6 levels *in vivo*. Here, we observed that single-dose minocycline treatment resulted in considerable decrease in plasma (Fig. 8A) and tumor (Fig. 8B) IL-6 levels after both 4 and 24 h. Furthermore, tumoral expressions of p-STAT3 and MCL-1 were significantly inhibited in 4 and 24 h treatment samples. Inhibition of the ERK1/2 activation was observed in the 4 h treated tumor samples which consistent with the *in vitro* results, seems to be recovered by 24 h (Fig. 8B).

## Discussion

Here, we report for the first time the modulatory effect of minocycline on IL-6 system in ovarian cancer. We first demonstrated the inhibition of both constitutive and IL-1ß or 4-OH-E2 stimulated IL-6 expression by minocycline in ovarian cancer cell lines *in vitro*. This was followed by acute treatment of ovarian tumor bearing mice with minocycline for 4 and 24 h. Analysis of plasma and tumors collected from these mice revealed down-regulation of IL-6 protein in both plasma and tumors. These results are in accordance with previous studies showing the inhibitory effect of minocycline on IL-6 expression in central nervous system which has accredited minocycline as an important neuroprotective agent [20], [21]. In addition to inhibiting IL-6 expression, minocycline also profoundly inhibited two major components of IL-6 receptor system namely IL-6Rα and gp130. Suppression of IL-6, down-regulation of IL-6Rα coupled with reduced gp130 expression may basically protect cells against IL-6 or sIL-6R assault. It has been shown that interference with either IL-6Rα or gp130 would negatively impact IL-6 biological effects [39], [40]. Therefore, it seems probable that even in the presence of excess amount of IL-6 minocycline may still have its inhibitory effect on IL-6 system. Based on the detrimental consequences of IL-6 activity in ovarian cancer including induction of chemoresistance and malignant ascites formation, targeting IL-6 and its receptor system is considered as an emerging therapeutic approach for cancer chemotherapy. The promising results obtained from pre-clinical and clinical studies evaluating anti-IL-6 antibodies in recurrent chemotherapy-resistant ovarian cancer are a strong supportive proof for this claim [3], [41]. In addition, while immunologic responses have been a draw-back in some of the studies using anti-IL-6 monoclonal antibodies [42], minocycline as a non-biologic agent is not expected to cause such responses. We recently reported that orally administered minocycline led to suppression of tumor growth and proliferation index and conferred survival advantage in female nude mice bearing OVCAR-3 xenografts [27]. We have also shown that minocycline inhibited ovarian cancer induced malignant ascites formation in mice. In view of the capacity of minocycline to suppress tumor growth and malignant ascites formation, the minocycline-mediated blockade of IL-6 and its receptor system may be considered as an important contributing mechanism.

A major pathway through which IL-6 triggers intracellular response is the activation of STAT3, a key point of integration between IL-6 and its tumorogenic effects. The IL-6-STAT3 signaling pathway plays a significant role in development and progression of ovarian cancer. It has been shown that the phosphorylation and translocation of STAT3 to the nucleus are frequent events in ovarian carcinoma that are associated with poor prognosis [43]. While several reports recognize STAT3 as an oncogenic factor in ovarian cancer, interestingly there are reports showing that it may play a dual role in some other cancers where it acts as a tumor-suppressor [44], [45] or correlates inversely with metastasis [46], [47]. However, in ovarian cancer it is known that tyrosine phosphorylation is mainly associated with oncogenic status of STAT3. In our study, minocycline inhibited Tyr-705 phosphorylation of STAT3 in both non-stimulated and IL-1β-stimulated cells. However, this effect was faded away by 24 h. It seems that cells manage to compensate and the level of phosphorylated STAT3 returns to control value by 24 h due to the cellular turnover. In support of the *in vitro* results, the same inhibitory effect on Tyr-705 phosphorylation of STAT3 was also observed in tumors excised from mice at 4 and 24 h post minocycline treatment. Since IL-6 is known to be the major cytokine fuelling STAT3 activation in ovarian cancer cells [48], the inhibitory effect of minocycline on STAT3 activation may be attributed to its IL-6 modulatory effects. Our results are in line with previous reports demonstrating that blockade of IL-6 by its selective antibody inhibits STAT3 phosphorylation in ovarian cancer cells [41], [49].

Unphosphorylated STAT3 resides in the cytoplasm. After Tyr-705 phosphorylation, STAT3 dimerizes and translocates into the nucleus [50]. Ovarian tumor tissues have activated STAT3 in the nucleus and not in the cytoplasm [43]. Here we have shown that the nuclear translocation of STAT3 is substantially reduced by minocycline treatment. Inhibition of STAT3 nuclear translocation in turn inhibits transcriptional activation of various gene products that are regulated by STAT3. These include the survival protein Mcl-1 [51]. Mcl-1 is a member of the Bcl-2 family of apoptosis-regulating proteins. In recent years, it is increasingly being realized that over-expression of Mcl-1 contributes to cancer progression and confers resistance to apoptosis [52]. In our study, Mcl-1 expression was down-regulated in both unstimulated and IL-1β-stimulated cells *in vitro* as well as in tumors excised from minocycline-treated mice at both 4 and 24 h *in vivo*. The blockade of Mcl-1 expression by minocycline may at least partly be accounted for by the minocycline-induced IL-6/STAT3 inhibition. These observations confirm previous studies indicating that inhibition of IL-6/STAT3 axis down-regulates MCL-1 in ovarian cancer [41], [53].

Autocrine production of IL-6 in epithelial ovarian cancer cell lines is positively associated with activation of MEK/ERK [54]. Using genetically altered mouse ovarian cancer cell lines and tumors has proved the functional contributions of ERK1/2 to ovarian cancer development and maintenance [55]. Here we have shown that minocycline transiently blocks activation of ERK1/2 in IL-1β-stimulated ovarian cancer cells which rapidly is compensated due to the known rapid turnover of p-ERK1/2 [56]. This inhibitory effect was also observed in tumors from mice that were euthanized after 4 h of minocycline administration. However, in agreement with our *in vitro* findings, no suppressive effect was observed in tumors excised from mice after 24 h of minocycline treatment. Hence minocycline not only interferes with the IL-6-STAT3 signaling but also affects the MAPK pathway which is amongst the most important oncogenic pathways. On the other hand, since activation of ERK1/2 is known as an indispensible step in IL-1β-stimulated up regulation of proinflammatory cytokines such as IL-6 [57], the inhibitory effect of minocycline on IL-6 surge after IL-1β stimulation might be explained by the observed suppressive effect of minocycline on ERK1/2 activation.

MAPK and STAT3 pathways are highly involved in tumor cell migration and invasion where their activation induces MMPs expression leading to the degradation of extracellular matrix proteins [38], [58]. MMP-2 and MMP-9 degrade collagen IV, the major structural collagen of the basement membrane, and are suggested to be critical in the metastatic process of ovarian cancer [59]. Here, we observed that minocycline suppressed multistep of ovarian cancer cell metastasis processes including migration, invasion and adhesion ability in a concentration-dependent manner which was coupled with down-regulation of MMP-2 and MMP-9 proteins. These results were consistent with the observed blockade of both IL-6/MAPK and IL-6/STAT3 pathways. Our results agree with previous studies showing that blocking IL-6 attenuates cancer cell invasiveness through down regulation of MMP-2 and MMP-9 expression [60], [61]. They are also in accordance with the reported suppressive effect of minocycline on MMP-2 and MMP-9 expression [62], [63]. What we have shown here strengthens the potential of minocycline as a multi-target drug in anticancer therapy.

In summary, the *in vitro* and *in vivo* data presented here demonstrate that minocycline exerts suppressive effects at multiple levels of the IL-6 signaling pathway in ovarian cancer cells. Furthermore, under cell culture conditions, minocycline attenuates cellular metastatic activity including, migration, invasion and adhesion. These results provide a rational basis for further evaluation of minocycline in the treatment of ovarian cancer and perhaps other IL-6-dependent malignancies.
