# Expression of Concern: Minocycline Suppresses Interleukine-6, Its Receptor System and Signaling Pathways and Impairs Migration, Invasion and Adhesion Capacity of Ovarian Cancer Cells: In Vitro and In Vivo Studies

**DOI:** 10.1371/journal.pone.0298444

**Published:** 2024-02-02

**Authors:** The PLOS ONE Editors

Following the publication of this article [1], concerns were raised regarding the results presented in multiple figures. Specifically,

- In Fig 5B, Lanes 1–2 of 2 h β-actin panel appear similar to Lanes 3–4 of the 24 h β-actin panel.
- The following β-actin panels appear similar:
  ○ β-actin for IL-6 and MCL-1 at 4 h in Fig 8B.
  ○ β-actin for IL-6 and MCL-1 at 24 h in Fig 8B.
  ○ β-actin at 2 h in Fig 4A and 4B.
  ○ β-actin at 6 h in Fig 4A and 4B.
  ○ β-actin at 24 h in Fig 4A and 4B.
- The following panels appear to overlap or partially overlap with images published in [2]:
  ○ ERK1/2 panel at 24 h in Fig 8B of [1] and lanes 1–4 of the p65 panel in Fig 5F of [2].
  ○ OVCAR-3 Control IL-6, PI and Merge panels in Fig 1 of [1] and OVCAR-3 Mino TGF-β1, PI and Merge panels in Fig 4A of [2] rotated.
  ○ SKOV-3 IL-6, PI and Merge panels in Fig 1 of [1] and SKOV-3 TGF-β1, PI and Merge panels in Fig 4A of [2] rotated.

**Fig 5:**
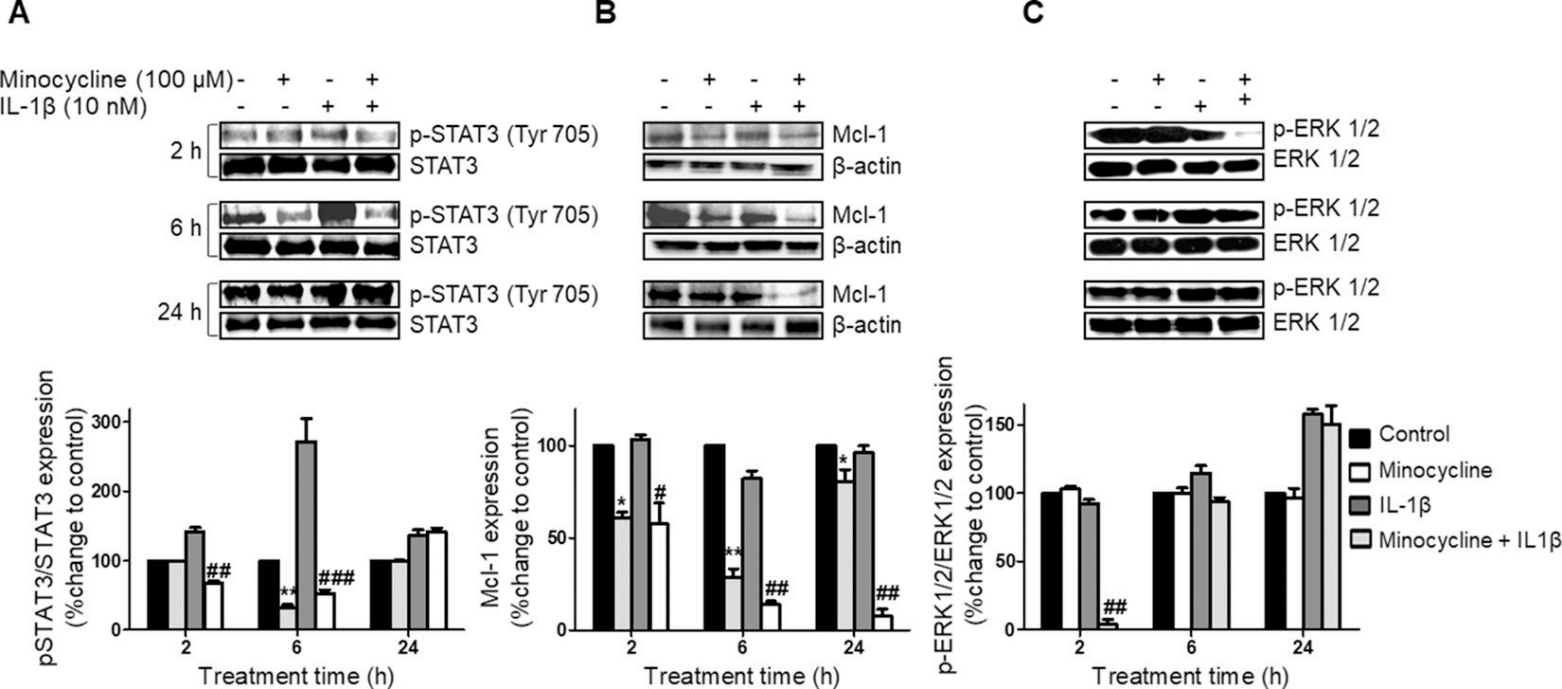
Influence of minocycline on p-STAT3, p-ERK1/2 and Mcl-1 expression in ovarian cancer cells. SKOV-3 cells were treated with minocycline (100 μM) with or without IL-1β (10 ng/ml) stimulation for different time points. The expression levels of (A) p-STAT3, STAT3; (B) Mcl-1 or (C) p-ERK1/2, ERK1/2 were estimated by western blot analysis. Densitometric analysis is expressed as mean ± SD intensity of optical density obtained by three independent experiments (*p<0.05, **p<0.01 and ***p<0.001 vs. control cells, ^#^*p*<0.05, ^##^*p*<0.01 vs. IL-1β treated cells).

The corresponding author stated that none of the original data underlying this article remain available.

The corresponding author acknowledged that the 24 h β-actin panel in Fig 5B is incorrect and provided an updated figure published with this notice. They clarified that the β-actin panels in Fig 4A and 4B and within Fig 8B were reused as the blots were stripped and reprobed. In the absence of original images underlying these figures, these issues cannot be fully resolved.

The corresponding author stated that images from this article [1] were inadvertently reused in the other article [2], and they asserted that the *PLOS ONE* article presents the correct data.

The *PLOS ONE* Editors issue this Expression of Concern due to the number of panels and figures affected and the absence of original data, which raise concerns regarding the overall reliability of the data presented in the original article.
